# Melatonin MT1 Receptor Expression in Luminal Invasive Ductal Breast Carcinoma in Postmenopausal Women

**DOI:** 10.3390/biom15040581

**Published:** 2025-04-15

**Authors:** Leda Pistiolis, Sahar Alawieh, Thorhildur Halldorsdottir, Anikó Kovács, Roger Olofsson Bagge

**Affiliations:** 1 Department of Surgery, Institute of Clinical Sciences, Sahlgrenska Academy, University of Gothenburg, 41390 Gothenburg, Sweden; roger.olofsson.bagge@gu.se; 2 Department of Surgery, Sahlgrenska University Hospital Gothenburg, 41345 Gothenburg, Sweden; 3 Department of Clinical Pathology, Sahlgrenska University Hospital Gothenburg, 41345 Gothenburg, Sweden; sahar.alawieh@vgregion.se (S.A.); aniko.kovacs@vgregion.se (A.K.); 4 Landspitali University Hospital, 105 Reykjavik, Iceland; thorhih@landspitali.is; 5 Wallenberg Center of Translational Medicine, University of Gothenburg, 41345 Gothenburg, Sweden

**Keywords:** melatonin, MT1 melatonin receptor, breast cancer

## Abstract

Laboratory and animal studies indicate that melatonin exerts a negative impact on breast cancer progression and metastasis. These actions are both receptor-dependent and -independent. Of the two transmembrane melatonin receptors identified in humans, breast cancer expresses only MT1. The aim of this study was to investigate the expression of MT1 in hormone-receptor-positive, HER2-negative invasive ductal breast carcinoma in postmenopausal women and its possible correlations with clinicopathological parameters and survival. A total of 118 patients with luminal A/B primary breast cancer with or without axillary metastases were identified. The MT1 receptor expression was immunohistochemically assessed as a percentage of stained cells and a weighted index (WI) (percentage multiplied by staining intensity). Most tumor samples (84.7%) and metastasized lymph nodes (96%) stained positive for MT1, with varying intensity. No statistically significant correlations were found between the MT1 expression or the WI in the primary tumor and the patient and tumor characteristics, or the MT1 and WI in the metastasized lymph nodes. The survival analysis did not reveal a significant effect of MT1 expression or the WI on the risk of recurrence or survival.

## 1. Introduction

Melatonin is a highly conserved molecule throughout evolution produced by all living organisms: bacteria, plants, and animals alike [1,2]. As evolution progressed and the complexity of organisms increased, it is speculated that its production was shifted from the mitochondria to the pineal gland, and its role expanded to include circadian rhythm regulation and sleep induction, as well as seasonal breeding. Its ancestral antioxidant capacities are still maintained and research has revealed additional functions that have rendered this ubiquitous molecule the focus of vigorous investigation: immunomodulation, neuronal protection, anti-aging, anti-inflammatory properties, anti-apoptotic properties in normal cells, and last but not least, anti-tumoral properties [3]. These actions are either receptor-independent or achieved via cellular receptors. The former are mainly due to melatonin’s antioxidant capabilities, with melatonin being produced and acting locally, mainly within the mitochondria [4]. The latter are mediated by receptors located primarily in the cellular membrane, although there are reports of cytosolic and even nuclear ones [5,6]. In humans, two types of membrane receptors have been identified: melatonin receptor 1 (MT1) and melatonin receptor 2 (MT2). Both are G protein-coupled transmembrane receptors with a high degree of homology (60%) [7,8]. But this is where their similarities end, since they are distinctly distributed among tissues where they serve diverse functions. This is further supported by the discovery of selective ligands for each of them [8].

Light exposure at night reduces melatonin production, and this has been suggested to increase the risk for breast cancer [9], with some preliminary reports appearing in the late 1990s. The first major study was the Nurses’ Health Study, with 78,562 participants and a 10-year follow-up in its initial publication. It showed a moderately increased risk for breast cancer in nurses working on night shifts [10]. Many similar studies followed, and a meta-analysis of 26 studies [11] showed a statistically significant relationship between short-term (<10 years) night shift working and breast cancer risk. However, in the studies of high quality, the risk was increased in both short- and long-term night shift work. In 2007, the International Agency for Research on Cancer classified night shift work as a probable carcinogen (Group 2A) [12].

Even though melatonin has intrigued the interest of researchers for several decades, and much has been discovered in the neuroendocrine front and its role as a circadian regulator, there are still conflicting issues concerning its additional functions in the organism. Its role as a free radical scavenger has been challenged due to a lack of sufficient experimental evidence [13,14], the validity of immunoassay kits used to measure the melatonin concentration has been put under the microscope [15], its role as an immunomodulator has been questioned [16], and melatonin production in the gastrointestinal tract has been revisited, contradicting previous results [17]. Melatonin receptor polymorphism has been implicated in type 2 diabetes [18] and certain cancers [19]. Moreover, it should be taken into consideration that many of the effects reported in preclinical studies were achieved in the μM to mM melatonin concentration range in the culture media [20], while the physiological concentration is in the nM range [21,22]. There is still much to be learned about the pharmacokinetics of melatonin in the human body, in both healthy and diseased states, including the dosage and the optimal mode of administration [23,24]. The existence of contradictions and gaps in our knowledge indicates that there is much yet to be determined and learned. In all fairness, one should be critical and even skeptical when it comes to the multiple roles that have been assigned to melatonin.

Breast tissue predominantly expresses MT1, but breast cancer cells exclusively express MT1. It is through them that the oncostatic action of melatonin on breast cancer cells is considered to take place [25]. And it is not just the exclusivity of MT1 in the tumor, but also the fact that its expression is several orders of magnitude larger than in normal breast tissue [26,27]. This has led to numerous studies [26,27,28,29,30] trying to establish a connection between MT1 and tumor biomarkers as well as patient characteristics. The findings of these studies do not agree for all parameters, leaving room for further exploration in order to uncover potential clinical significance and utility. MT1 expression has been studied in all breast cancer types and its association has been examined against multiple tumor characteristics: estrogen receptor (ER), progesterone receptor (PR), human epidermal growth factor receptor 2 (HER2), Ki67, and the tumor grade, to name a few. No single parameter has been unanimously correlated, whether positive or negative, to MT1 across all studies [26,27,28,29,30].

The “melatonin hypothesis” proposed by Cohen in 1978 [31] postulates that a decrease in melatonin production, as seen in postmenopausal women, causes a state of relative hyperestrogenism, which acts as a contributing factor for the development of breast cancer. Indeed, such a state can occur in some women during perimenopause, due to an imbalance between estradiol and progesterone production. Partial support for this hypothesis came from a study showing a decrease in the levels of plasma melatonin in women with hormone-receptor-positive breast cancer, which was proportional to the degree of hormonal positivity [32]. Melatonin production does decrease with age [33], as evidenced by insomnia and low sleep quality in the elderly.

We decided to investigate the MT1 expression in luminal, HER2-negative invasive ductal breast carcinoma in postmenopausal patients. The focus on postmenopausal patients was chosen in order to indirectly revisit the “melatonin hypothesis”. The present study aimed to investigate the relationship between MT1 expression and ER expression, PR expression, Ki67, tumor protein 53 (TP53), tumor size, tumor grade, lymph node status, the Nottingham prognostic index (NPI), and patient age. It also examined whether there was a relationship between MT1 expression and overall survival (OS), breast-cancer-specific survival (BCSS), and recurrence-free interval (RFI).

## 2. Materials and Methods

The expression of ER, PR, TP53, and Ki67 had already been determined post-operatively using standard immunohistochemistry, along with the tumor type, the size, and the presence of nodal metastases. HER2 assessment was conducted, first immunohistochemically and then via silver in situ hybridization. Consequently, an assessment of the abovementioned parameters was not repeated.

For the immunohistochemical assessment of MT1 receptors, four-micrometer-thick sections were made from formalin-fixed, paraffin-embedded blocks. The blocks were pretreated using the Dako PTLink system (Dako, Carpinteria, CA, USA) and processed further on an automated DAKO Autostainer platform. Melatonin antibodies (Abcam Cat# ab203346, RRID:AB_2783824, Cambridge, UK) (1:200) were used for melatonin receptor 1B/MTNR1B staining. Human pineal gland (corpus pineale) was used as a positive and negative control for antibody validation. The original melatonin immunohistochemical slides were scanned using a NanoZoomer S210/Hamamatsu (Oncotopix^®^ Scan by Visiopharm, Hoersholm, Denmark) at 40× magnification, and histological photos were taken from the digital images. All the slides were assessed by the same board-qualified pathologist subspecialized in breast pathology. The MT1 expression was rated both as the staining intensity (1: faint, 2: weak, 3: moderate, 4: strong) and the percentage of stained cells. The weighted index (WI) was calculated by multiplying the two scores.

For the survival analysis, the last day of follow-up was 1 December 2023, the date at which all patient files were reviewed. The OS was determined in months from the date of surgery to either the date of death, irrespective of the cause, or the last day of follow-up if the patient was still alive. The BCSS was determined in months from the date of surgery to either the date of death from breast cancer only, or the last day of follow-up if the patient was still alive. The RFI was determined in months from the day of surgery to either the date of recurrence, death from breast cancer, or the last hospital visit confirming that the patient was recurrence-free. Recurrence was defined as an invasive ipsilateral/contralateral breast/chest wall/axillary recurrence or a distant metastasis.

The *NPI* was calculated for each patient according to the standard formula [34]:

$$NPI=LN\;\left(0=1,\;1-3=2,>3=3\right)+Grade+0.2\times tumor\;size\;\left(cm\right)$$

A statistical analysis was conducted using the SPSS 29.0.1.1 statistical package (IBM Corp., Armonk, NY, USA). The Shapiro–Wilk test was used to determine if the variables were normally distributed, and Pearson’s correlation coefficient was used to determine any correlation between them. The Kruskal–Wallis test was used to investigate the differential expression of MT1 melatonin receptors and the WI according to the grade, and the Mann–Whitney U test was used to investigate the same variables in patients with metastasized and non-metastasized lymph nodes. A survival analysis was conducted using Cox regression and the enter method.

This study was approved by the Swedish Ethical Review Authority (Dnr: 479-18, 27-06-2018).

## 3. Results

This study was conducted on tumor samples from consecutive patients operated on at Sahlgrenska University Hospital, Gothenburg, Sweden, from 1 January 2003 to 31 December 2004. The inclusion criteria were defined as follows: postmenopausal status, invasive ductal breast carcinoma, ER positive, PR positive/negative, and HER2 negative. Patients who received neo-adjuvant treatment were excluded. A total of 153 patients were identified according to the inclusion and exclusion criteria. Of these, three patients were excluded because of bilateral breast cancer (*n* = 150). The paraffin-embedded specimens were retrieved from the paraffin block archive of the Department of Clinical Pathology, Sahlgrenska University Hospital. We were unable to recover the paraffin blocks of 30 patients (*n* = 120). During the assessment of the stained slides, two of them were excluded because of technical errors during preparation/staining, leaving a final 118 specimens to be used for the analysis. Forty patients of the original 153 patients also had an axillary metastasis. Paraffin blocks were retrieved for 28, of them and during the assessment, 1 slide was discarded due to a technical error, yielding 27 slides with nodal metastases. An additional 2 were removed because the paraffin block of the original tumor was not retrieved, leaving a total of 25 patients with paired axillary metastases. The patient and tumor characteristics are summarized in Table 1.

### 3.1. MT1 Receptor Expression

**Figure 1:**
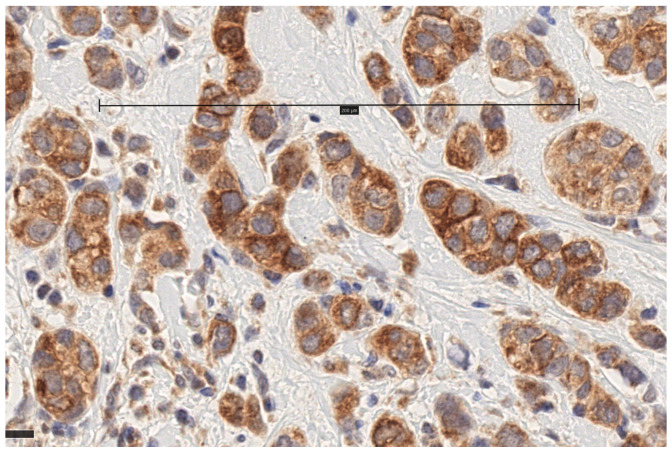
Invasive ductal carcinoma with combination of membrane and cytoplasmic staining (scale bar: 200 μm).

**Figure 2:**
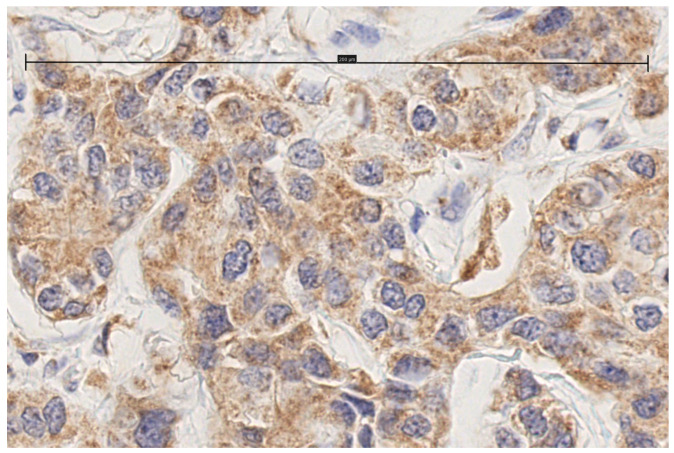
Invasive ductal carcinoma with moderate cytoplasmic staining (scale bar: 200 μm).

**Figure 3:**
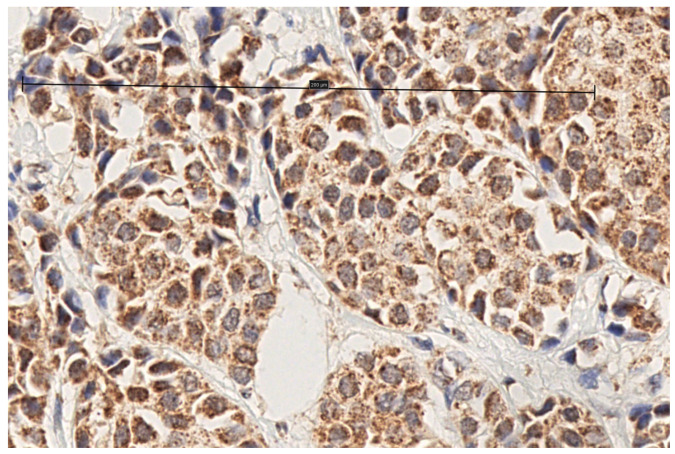
Invasive ductal carcinoma with strong cytoplasmic staining (scale bar: 200 μm).

**Figure 4:**
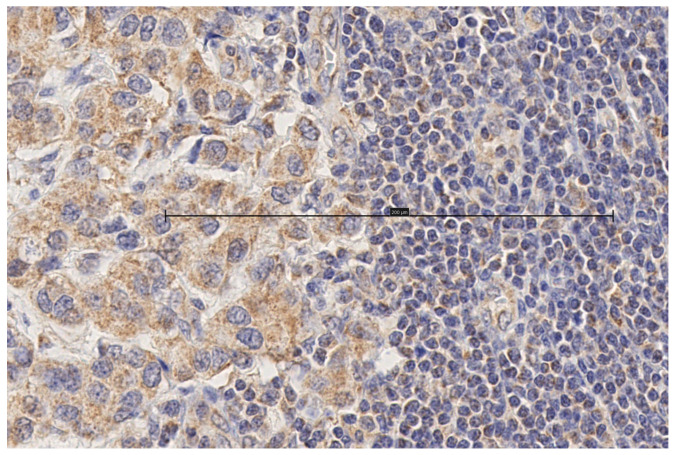
Lymph node metastasis. Invasive ductal carcinoma with weak cytoplasmic staining (scale bar: 200 μm).

Immunohistochemical staining for MT1 receptors was noted in 100 of 118 (84.7%) tumors and 24 of 25 (96%) metastasized lymph nodes. In the cases of positive IHC expression, there was either cytoplasmic staining or combined cytoplasmic and membrane staining (Figure 1, Figure 2, Figure 3 and Figure 4, scale bar: 200 μm).

**Figure 5:**
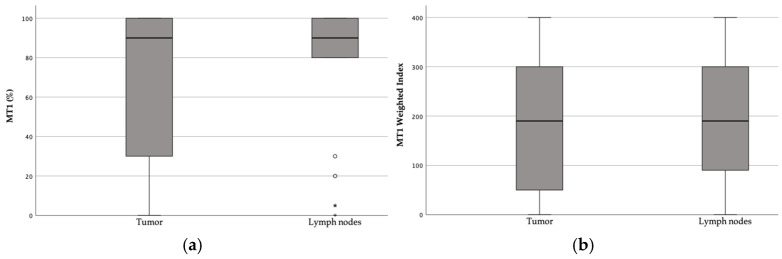
Box plots of the MT1 expression (**a**) and weighted index (**b**) in the tumor and metastasized lymph nodes.

The melatonin receptor expression in the tumors (*n* = 118) ranged between 0 and 100%, with a median of 90% (IQR: 30–100). The tumor WI ranged between 0 and 400, with a median of 190 (IQR: 38–300) (Figure 2). In the assessed metastatic lymph nodes (*n* = 25), the MT1 expression ranged from 0 to 100% with a median of 90% (IQR: 80–100), and the WI ranged from 0 to 400 with a median of 190 (IQR: 85–300) (Figure 5).

### 3.2. Correlation of MT1 Receptor Expression and WI with Patient and Tumor Characteristics

The correlation of the tumor MT1 receptor expression and the WI with the patient and tumor characteristics was analyzed using Spearman’s correlation coefficient. No significant correlation was detected for the patient age, tumor size, ER, PR, ki67, TP53, NHG, or NPI and MT1 nodal expression (Table 2). The WI of the tumor was similarly analyzed for correlations with all the above-mentioned parameters, with the exception of MT1 in the nodes, which was replaced by WI in the nodes. Again, no significant correlation was found (Table 3).

There was no difference in the distribution of the tumor MT1 and WI among the different histological grades (Kruskal–Wallis test, *p* = 0.640 and 0.196, respectively). There were no statistically significant differences in the median tumor MT1 and WI among patients with or without metastases (Mann–Whitney U test, *p* = 0.776 and 0.702, respectively).

### 3.3. Survival Analysis

On the last day of follow-up (1 December 2023), 73 out of 118 (61.9%) patients had died, 15 of which (20.6%) died due to breast cancer. Nine patients (7.7%) had a locoregional recurrence, three had contralateral breast cancer (2.5%), and seventeen (14.4%) developed distant metastases. The median OS was 182 months and the median BCSS was not reached.

A univariate Cox regression analysis was performed to identify significant factors for the OS, BCSS, and RFI. The following parameters were examined: the patient age, tumor size, NHG, ER, PR, ki67, Tp53, N status, NPI, MT1 tumor, and WI tumor. The only parameter found to be statistically significant for the OS was the patient age (HR: 1.096, 95% CI: 1.067–1.126, *p* < 0.001) (Table 4). The significant prognostic factors for the BCSS included the tumor size (HR: 1.030, 95% CI: 1.004–1.056, *p* = 0.022), Ki67 (HR: 1.029, 95% CI: 1.008–1.050, *p* = 0.006), and NPI (HR: 2.803, 95% CI: 1.724–4.558, *p* < 0.001) (Table 4). For the RFI, the significant prognostic factors included the tumor size (HR: 1.032, 95% CI: 1.010–1.054, *p* = 0.004), Ki67 (HR: 1.020, 95% CI: 1.000–1.039, *p* = 0.046), N1 status (HR: 29.767, 95% CI: 3.013–294.085, *p* = 0.004), N2 status (HR: 24.332, 95% CI: 1.412–419.289, *p* = 0.028), and NPI (HR: 2.450, 95% CI: 1.667–3.602, *p* < 0.001) (Table 4).

In the multivariate Cox regression analysis for the BCSS and RFI, adjusting for the patient age and NPI did not reveal any statistically significant effects on the MT1 expression (Table 5) or WI (Table 6).

## 4. Discussion

The research conducted so far on the relationship between MT1 and the tumor characteristics in patients with breast cancer has delivered conflicting results, thus failing to create a firm foundation on which future investigations can be based. The present analysis could not evade this norm, as it failed to identify any correlation between MT1 receptor expression and common patient and tumor characteristics, recurrence, or survival. Previous studies have included all types of breast cancer [27,28] together with breast cancer cell lines [26,29]. One study [30] concentrated on triple negative breast cancer only. The present discussion focuses primarily on the findings pertaining to hormone-receptor-positive breast cancer, unless stated otherwise.

In accordance with previous studies, we observed both cytoplasmic and a combination of cytoplasmic and membrane IHC staining for the MT1 receptor [26,27,28,29]. Melatonin receptor expression was found to be uniformly higher in cancerous compared to normal breast tissue. There appeared to be an up-regulation of the receptors in breast tumors, and the explanation for this could be twofold. First, it has long been known that serum melatonin levels are lower in women with breast cancer compared to those without [35], and this decrease is inversely proportional to ER and PR positivity [36]. In other words, a decrease in serum melatonin due to the presence of a tumor is further accentuated by the hormonal positivity of the tumor, leading to increased receptor transcription. In addition, the patients in our cohort were women aged 56–89 years, and melatonin production is known to decrease with age [37,38]. Second, the increased number of free radicals produced during aerobic glycolysis in cancer cells increases the demand for antioxidants within the cell [39], and therefore, the need for melatonin. Nevertheless, even though MT1 expression has been reported to be higher in hormone-receptor-positive breast cancer as compared to triple negative, there seems to be a disagreement concerning the relationship between melatonin and estrogen receptor expression in those tumors. The studies conducted so far have demonstrated no significant correlation [28] or a statistically significant inverse correlation [29] between MT1 and ER. Our findings agree with the first, with no association. In a similar fashion, the expression of PR is either not correlated [26,28] or inversely correlated [29] with MT1 expression. Our findings here also agree with the first, with no association. Patient age was not associated with MT1 expression in three studies [26,28], including this one, but one study [27] demonstrated an approximate 30% increase in the immunoreactive score for the receptor in the 41–60 age group, compared to 31–40, and a 50% decline after the age of 61. It was not stated, however, if these differences were found to be significant. Lymph node status was not found to be significant in any of the studies.

Of interest are the findings concerning the tumor grade and Ki67. Both a significant correlation [30] between increasing MT1 expression and an increasing grade and a predilection for an inverse correlation with statistical significance for grades II and III [26] have been reported. Our analysis did not reveal any correlation. Statistically significant negative correlations between Ki67 and MT1 and between Ki67 and the WI have been reported, with the latter in triple negative breast cancer [26,30]. Our findings showed no association, and this is in accordance with other studies [27,29]. Given the fact that the tumor grade and Ki67 are closely correlated [40], this finding deserves further investigation. It could be hypothesized that, as cancer cells lose their differentiation and progress into a more advanced grade with increased proliferation, their ability to synthesize MT1 decreases. Or it could be that receptor transcription is downregulated by melatonin itself [29] through increased influx from circulation or augmented intracellular production to meet the increased metabolic demands.

The survival analysis did not show any prognostic value of MT1 expression in ER-positive tumors, as has been shown for patients with triple negative breast cancer [30]. Furthermore, no association was found between MT1 expression and known prognostic factors such as the patient age, lymph nodal status, tumor grade, and NPI. The follow-up of the patients was long enough, both to allow for the long relapse time of luminal breast cancers [41,42], especially luminal A, and to draw safe conclusions. Consequently, the potential use of MT1 in hormonal-positive breast cancer as a prognostic factor appears to be limited.

The study does contain some limitations. Only invasive ductal carcinoma was included, but this decision was made because it represents the most common type of breast cancer [43]. HER2-positive tumors were excluded, even though they have been found to express MT1 [26,27,28]. Some could argue that the patient characteristics represent a limitation per se, but the aim of this study was very specific, since we wanted to explore the Cohen hypothesis. Furthermore, the number of samples selected could represent a limitation in the sense that it was not large enough to detect a true difference. On the other hand, however, it was large enough to depict a trend between the variables in question. Last, but not least, since MT1 immunohistochemistry is not included in routine pathological assessments, the experience is limited. As a counterbalance for this, we tested several different antibodies with both positive and negative controls before choosing the definitive antibody that was used to analyze all the samples. The one that was finally chosen was the only one that worked with both positive and negative controls.

Contrary to the general belief, approximately 95% of the body’s total melatonin production is not produced by the pineal gland [44,45]. As early as 1974, shortly after the discovery of melatonin, Cardinalli et al. provided evidence of melatonin synthesis in the retina, and succeeding research confirmed its production in almost all mammalian tissues [4]. Local production is believed to take place in the mitochondrial matrix, serving the primordial role of melatonin, namely that of an antioxidant/antioxidant inducer against the free radicals generated during cellular respiration [1,13,22,46]. Moreover, in situ melatonin production is hypothesized to be, at least in part, regulated by local demand [47]. The above holds true for normal functioning cells. Cancer cells, however, exhibit an altered metabolism, which was first described by O. Warburg, who observed that cancer cells prefer anaerobic glycolysis to oxidative phosphorylation, even in the presence of sufficient oxygen [48]. This came to be known as the Warburg effect, and in diseased cells, it is attributed to defective mitochondrial function. One of the repercussions of this deranged metabolism is the reduced influx of pyruvate in the mitochondria, resulting in decreased local melatonin production [49], which has also been confirmed experimentally. The above could have therapeutic implications, especially for hormone-receptor-positive breast cancers, which exhibit melatonin receptors.

In a recent systematic review [50] on melatonin’s actions in breast cancer cell lines and animal experiments, it was concluded that melatonin exerts multiple effects against tumor progression and metastasis, by promoting the apoptosis and autophagy of cancer cells and inhibiting angiogenesis. Cell cycle arrest is an additional oncostatic effect. These actions are receptor-dependent, but can also be due to direct action in the cytosol or the nucleus [51]. A cytosolic receptor, formerly called MT3 [52] and later identified as the human homologue of quinone reductase II, has also been implicated in the oncostatic effects of melatonin, principally through its antioxidant functions [53,54], and there are research data to support its potential use in the clinical setting [55]. Furthermore, the interplay between melatonin and estrogen has been known for a long time. After all, melatonin plays a pivotal role in the initiation of puberty. In the case of breast cancer, it acts both as a selective estrogen receptor modulator (like tamoxifen) and as a selective enzyme receptor modulator (like aromatase inhibitors) [56] in order to decrease the influx of estrogens in the tumor cell [57]. Moreover, it has been shown that melatonin can decrease the expression of ER in breast cancer cells [58], thus reducing estrogen-induced proliferation. Taking the above into consideration, one can see potential uses of melatonin in oncology, as an adjunct to already existing therapies. Two meta-analyses [59,60] on clinical trials with the addition of melatonin to the patients’ oncological treatment (various types of stage IV cancers) showed that patients in the melatonin arm demonstrated a reduction in the risk of death and a significant improvement in complete and partial remission. A decrease in the toxicity of the chemotherapy was an additional benefit. Two studies have been conducted solely with breast cancer patients with progressing/non-responsive stage IV breast cancer [61,62]. The first was a randomized trial with 40 patients with ER-negative tumors, too frail to undergo chemotherapy, and progressing on tamoxifen alone. The second was a cohort study of 14 patients progressing on tamoxifen, where melatonin was added to the therapeutic regimen and each patient acted as her own control. Both studies were small, but they do depict an improved outcome in relation to the response to treatment in the melatonin arm. Moreover, adding melatonin to culture media of breast cancer cell lines has been shown to synergistically act with chemotherapeutic agents [63,64], potentiating their effects. The pretreatment of breast cancer cells with melatonin also potentiates the effect of ionizing radiation [65,66]. In addition, even though one might think that the same cell growth inhibitory effects could be seen on normal breast tissue, they appear to be very selective, pertaining only to the tumor [67]. Melatonin agonists and a conjugate of melatonin with tamoxifen have already been tested in laboratories with encouraging results [68,69].

Melatonin is available over the counter as a dietary supplement in many countries, while a prescription is required in some others. In the adult population, it is primarily used as a sleeping aid. Numerous studies have been conducted that have reported on the adverse effects of melatonin in the participants. The two systematic reviews that have been published so far [70,71] concluded that melatonin administration appears to be safe for short-term use, with most of the reported adverse events being drowsiness, headaches, a reduction in psychomotor and neurocognitive function, and fatigue, especially when administered during the day. A decrease in heart rate and blood pressure has been reported in patients with cardiovascular problems; it is not clear whether this should be attributed to melatonin per se, or its interaction with antihypertensives. Effects on the endocrine system and glucose metabolism were also noted. All of these warrant further investigations, especially with the widespread use of melatonin. Furthermore, no studies on its long-term use have been conducted [14,72]. Another area of concern is elderly patients, who are found to exhibit delayed melatonin metabolism and excretion, leading to high plasma levels [73]. In addition, melatonin ingestion is not recommended during pregnancy and lactation [14].

The low cost and the relatively few adverse effects make melatonin an attractive adjuvant candidate for breast cancer treatment. A question that arises, however, is in which context and for which group of patients it could be applicable. It is this question that ours and similar studies are trying to answer. The studies conducted so far have failed to give concordant results regarding the relationship between melatonin receptors and the biological parameters of breast cancer. Based on the existing data, MT1 cannot be used as a prognostic factor, at least not in the hormonal-positive spectrum of the disease, but the presence of melatonin receptors on tumors could be exploited to the patients’ advantage. The induction of MT1 receptors by valproic acid in both ER-positive and triple negative breast cancer cell lines and their synergistic effect against cellular proliferation are a promising direction [30,74,75,76]. Future research should focus on in vivo studies to investigate the best way in which we can transfer the knowledge we have gained so far, from bench to bedside. One such trial, investigating the effect of melatonin on uveal melanoma, is currently under way [77].

## 5. Conclusions

As a final point, this study did not generate any significant correlations between the MT1 expression or WI in primary tumors or metastasized lymph nodes and the clinicopathological parameters examined. In addition, there was no prognostic value for the OS, BCSS, or RFI. The role of melatonin in breast cancer is still under investigation, driven by preclinical data and in combination with its relatively safe administration profile and low cost. More research is needed to determine if, and in what way, MT1 expression could be used as a prognostic or predictive factor and the framework in which data from research in breast cancer cell lines can be applied within the therapeutic context.

## Figures and Tables

**Table 1:** Patient and tumor characteristics.

		Median (IQR)	Patients (%)
Age		66 (61–79)	
Breast surgery	Mastectomy		45 (38.1%)
	Breast conservation		73 (61.9%)
Axillary surgery	Not performed		18 (15.3%)
	SLNB only ^1^		35 (29.7%)
	ALND ^2^		65 (55.1%)
Tumor size (mm)		17 (11–26)	
NHG	Grade I		40 (33.9%)
	Grade II		55 (46.6%)
	Grade III		23 (19.5%)
ER (%)		100 (100–100)	
PR (%)		70 (20–90)	
Ki67 (%)		10 (9–15)	
TP53 (%)		0 (0–10)	
Axillary nodal status	Nx		18 (15.3%)
	N0		67 (56.8%)
	N1		21 (17.8%)
	N2		5 (4.2%)
	N3		7 (5.9%)
NPI		3.39 (3.14–4.56)	

^1^ Two patients with a positive SLNB did not receive an ALND. ^2^ Seven patients with a positive SLNB received an ALND. (IQR: inter-quartile range, SLNB: sentinel lymph node biopsy, ALND: axillary lymph node dissection).

**Table 2:** Correlations for tumor MT1.

Variable	Spearman’s r	*p*
Age	0.073	0.433
Tumor size	0.416	0.416
ER	−0.132	0.157
PR	−0.037	0.693
Ki67	0.101	0.278
TP53	0.069	0.465
NHG	0.037	0.691
NPI	−0.016	0.874
MT1 nodes	−0.004	0.986

**Table 3:** Correlations for tumor WI.

Variable	Spearman’s r	*p*
**Age**	0.084	0.368
**Tumor size**	0.137	0.138
**ER**	−0.111	0.233
**PR**	−0.054	0.563
**Ki67**	0.154	0.096
**TP53**	0.034	0.718
**NHG**	0.105	0.644
**NPI**	0.046	0.644
**WI nodes**	−0.100	0.635

**Table 4:** Univariate Cox regression analysis for OS, BCSS, RFI. Statistically significant values are in bold.

	OS		BCSS		RFI	
	HR (95% CI)	*p*	HR (95% CI)	*p*	HR (95% CI)	*p*
Patient age	1.096 (1.067–1.126)	**<0.001**	1.052 (0.993–1.113)	0.084	1.016 (0.971–1.063)	0.495
Tumor size	1.015 (1.000–1.030)	0.057	1.030 (1.004–1.056)	**0.022**	1.032 (1.010–1.054)	**0.004**
NHG						
Grade I	Ref	0.068	Ref	0.175	Ref	0.126
Grade II	0.812 (0.490–1.344)	0.417	6.929 (0.886–54.166)	0.065	3.593 (1.032–12.509)	**0.044**
Grade III	0.807 (0.410–1.588)	0.534	6.851 (0.764–61.406)	0.085	3.366 (0.803–14.099)	0.097
ER	1.016 (0.995–1.036)	0.138	1.019 (0.972–1.068)	0.433	1.031 (0.978–1.087)	0.255
PR	1.004 (0.998–1.011)	0.194	0.999 (0.985–1.012)	0.846	0.997 (0.986–1.008)	0.593
Ki67	0.998 (0.983–1.013)	0.804	1.029 (1.008–1.050)	**0.006**	1.020 (1.000–1.039)	**0.046**
TP53	0.987 (0.973–1.002)	0.094	0.981 (0.941–1.022)	0.362	0.969 (0.924–1.018)	0.211
N status						
N0	Ref	0.402	Ref	0.584	Ref	**0.029**
N1	1.065 (0.312–3.637)	0.921	4.920 (0.307–78.812)	0.260	29.767 (3.013–294.085)	**0.004**
N2	3.491 (0.770–15.831)	0.105	0.000 (0.000–.)	0.996	24.332 (1.412–419.289)	**0.028**
N3	0.755 (0.174–3.271)	0.707	6.066 (0.379–97.190)	0.203	7.595 (0.466–123.670)	0.154
NPI	1.162 (0.934–1.445)	0.179	2.803 (1.724–4.558)	**<0.001**	2.450 (1.667–3.602)	**<0.001**
MT1 tumor	1.001 (0.996–1.008)	0.624	1.001 (0.988–1.014)	0.877	1.002 (0.991–1.013)	0.728
WI tumor	1.000 (0.998–1.002)	0.851	1.000 (0.998–1.002)	0.919	1.000 (0.997–1.003)	0.903

**Table 5:** MT1 tumor Cox regression analysis, adjusting variables for BCSS and RFI. Statistically significant values are in bold.

	BCSS		RFI	
	HR (95% CI)	*p*	HR (95% CI)	*p*
Patient age	1.053 (0.990–1.120)	0.099	1.036 (0.962–1.115)	0.351
NPI	2.657 (1.659–4.258)	**<0.001**	1.572 (0.772–3.199)	0.212
MT1 tumor	0.999 (0.984–1.013)	0.844	1.001 (0.981–1.022)	0.929

**Table 6:** WI tumor Cox regression analysis, adjusting variables for BCSS and RFI. Statistically significant values are in bold.

	BCSS		RFI	
	HR (95% CI)	*p*	HR (95% CI)	*p*
**Patient age**	1.055 (0.991–1.122)	0.093	1.035 (0.965–1.110)	0.330
**NPI**	2.688 (1.665–4.340)	**<0.001**	1.567 (0.772–3.182)	0.214
**WI tumor**	0.999 (0.995–1.004)	0.731	1.000 (0.996–1.005)	0.830

## Data Availability

The data are available from the corresponding author upon reasonable request.
